# A biphasic astrocytic PTGDS trajectory marks a metabolic vulnerability stage in prodromal Alzheimer’s disease

**DOI:** 10.21203/rs.3.rs-9499795/v2

**Published:** 2026-06-10

**Authors:** YoungOuk Kim, WooMyung Heo, Se Jin Park, YoungChul Kim, Ye Eun Cho, Ye-Won Lee, JungYeon Kim

**Affiliations:** 1 BioXP Research Institute, Donghae, Gangwon-do, Republic of Korea; 2 Department of Food Biotechnology and Environmental Science, Kangwon National University, Chuncheon 24341, Republic of Korea; 3 Bio R&D Team, Zefit Inc., Daegu, Republic of Korea

**Keywords:** Alzheimer’s disease, astrocyte, PTGDS, neuropathological transition, MCI, lipocalin-2, NGFR, metabolic inflection, SEA-AD, CSF biomarker, aging brain, cognitive aging

## Abstract

Alzheimer’s disease shows prolonged prodromal stability before accelerating decline, yet molecular markers resolving this heterogeneity are limited. Using pseudo-progression analysis of 1.3 million SEA-AD single nuclei (84 donors), we identify a reproducible biphasic astrocytic trajectory anchored to prostaglandin D2 synthase (PTGDS), with a statistically resolved donor-level inflection (quadratic β_2_ = −2.27, p = 0.006; vertex CPS 0.47). The same directional change is independently reproduced in external brain proteomics (ROSMAP and Banner; AD versus control p = 3.4 × 10^−3^), and the biphasic pattern reconciles previously conflicting CSF reports as stage-dependent. In ADNI CSF, downstream NEFL tracks cognitive decline strongly and LCN2 weakly, whereas PTGDS itself is tissue-restricted and not a stand-alone predictor. We propose, but do not establish, that post-inflection PTGDS attenuation accompanies LCN2-linked inflammation and NGFR suppression. These data position astrocytic PTGDS as a candidate stage marker, not a causal driver, of the compensatory-to-vulnerable shift in the aging brain — the astrocytic PTGDS inflection (CPS 0.47).

## Introduction

Mild cognitive impairment (MCI) represents a pivotal yet poorly resolved stage in Alzheimer’s disease (AD) progression and one of the most consequential transition points in the aging brain. Clinically defined as a prodromal state preceding dementia, annual MCI-to-AD conversion rates span 5–39%, and a substantial proportion of individuals remain stable or revert to normal cognition [1–3]. This heterogeneity implies that MCI does not represent a single biological state but rather a spectrum of substates differing fundamentally in reversibility and pathological commitment, and identifying the molecular event that distinguishes resilient cognitive aging from commitment to neurodegeneration is central to understanding how the aging brain transitions toward irreversible dysfunction. Existing staging frameworks based on amyloid burden, tau phosphorylation, and clinical cognitive scores [4,5] fail to resolve reversible compensation from progressive neurodegenerative commitment at the molecular level. The central unresolved question in prodromal AD is therefore the identification of a *transition boundary* — the molecular event that separates reversible compensatory states from irreversible neurodegeneration.

The limited disease-modifying efficacy of amyloid-targeted therapies [6,7] has redirected attention toward upstream events preceding overt neurodegeneration. Evidence increasingly implicates glial metabolic remodeling and impaired neurogenic support as determinants of neuronal instability that emerge before structural neuronal loss [8–15], raising the possibility that the critical disease transition is metabolic rather than proteopathic in origin. Neuroinflammatory activation alone does not adequately explain this transition, as inflammatory markers are elevated across the MCI spectrum without reliably distinguishing stable from progressive cases. A biologically meaningful transition boundary would therefore require a threshold-like molecular event capable of collapsing accumulated compensatory capacity and initiating downstream amplification cascades.

The SEA-AD atlas (Gabitto et al., 2024) [16], comprising 1.3 million nuclei from 84 donors aligned along a validated continuous pseudo-progression score (CPS), provides an unprecedented framework to interrogate this question. Within this dataset, astrocytic trajectories reveal a biphasic pattern characterized by early compensatory activation followed by abrupt attenuation at intermediate disease stages. Whether this inflection constitutes a true neuropathological transition boundary — defined by non-linear dynamics, statistical threshold behavior, and cross-species conservation — or reflects continuous gradual drift has remained unresolved.

Among candidate molecular mediators, lipocalin-2 (LCN2), an NF-κB-regulated inflammatory amplifier elevated in MCI and AD [17–19], links astrocytic dysfunction to synaptic instability and impaired CREB/NGFR-associated neurogenic signaling [20–25]. Upstream of this cascade, prostaglandin D_2_ synthase (PTGDS) represents a compelling astrocytic regulator. PTGDS integrates inflammatory tone with neurogenic support [26–28] and functions as a major amyloid-β chaperone [29], placing it at the convergence of metabolic buffering and neuroprotection. Among astrocyte-enriched transcripts in the SEA-AD atlas, PTGDS demonstrated the most pronounced biphasic inflection and the highest astrocyte-selective expression ratio within CPS 0.3–0.5, distinguishing it from other reactive astrocytic markers including CLU, GFAP, and AQP4. Conflicting human CSF data — PTGDS elevation in some cohorts [30,31] and reduction in others [32] — are consistent with stage-dependent biphasic dynamics obscured by cross-sectional sampling.

Here, we test the hypothesis that astrocytic PTGDS marks a statistically resolved metabolic inflection during the MCI-to-AD continuum that demarcates a regime of accelerated vulnerability. Using an integrated framework comprising (1) segmented regression and quadratic modeling within SEA-AD single-cell pseudo-progression, (2) a reversible zebrafish MCI model enabling temporal dissection of compensatory failure, (3) murine perturbation systems for mechanistic validation, and (4) longitudinal ADNI CSF proteomics, we identify a lipid-metabolic inflection at CPS 0.47 that marks the onset of accelerated vulnerability, identify PTGDS as its molecular anchor, and characterize a pre-inflection window of pharmacological responsiveness relevant to stage-stratified intervention.

## Materials and methods

### Ethical approval and animal husbandry

All mouse experiments were approved by the IACUC of Kangwon National University (Approval No. KW-241104–1) and conducted under controlled environmental conditions (23 ± 2°C, 50 ± 10% humidity, 12 h light/dark cycle). All zebrafish experiments were performed at Zefit Inc., an FDA-registered CRO (Approval No. ZEFIT-IACUC-26010601–0001). Zebrafish were maintained at 28.5°C, 14:10 h light:dark cycle, pH 7.0–7.5. Human dataset analyses utilized de-identified secondary data from public repositories (SEA-AD, ADNI); no additional IRB approval was required.

### SEA-AD snRNA-seq trajectory analysis

We analyzed 1.3 million nuclei from the SEA-AD middle temporal gyrus atlas [16] (quality control: ≥500 genes/nucleus, <20% mitochondrial reads; Scrublet-based doublet removal [33]). All donors derive from a uniformly aged human cohort (median age at death 90 years, range 65–102), and CPS is a continuous neuropathology-derived disease-progression score [16] rather than a chronological-age index; the trajectories analyzed here therefore resolve disease-stage dynamics within an aged baseline. CPS values were rounded to one decimal to define nine discrete progression bins (Bins 0.1–0.9), approximately aligned to Braak stages: Bin 0.0–0.3 (~Braak I–II), Bin 0.4–0.6 (~Braak III–IV), Bin 0.7–0.9 (~Braak V–VI). This mapping is heuristic and does not imply direct pathological equivalence. LCN2 showed an extremely low detection rate (0.007%) and was independently validated by orthogonal CSF proteomic and cross-species qPCR platforms (detailed in Results). Quality control metrics and bin-to-Braak stage mapping are detailed in Supplementary Table S1.

### Trajectory smoothing and cross-correlation analysis

Bin-level means were computed from log-normalized expression per cell type and smoothed using a 3-bin centered moving average to mitigate stochastic dropout. Lagged cross-correlation functions (ccf() in R, lag.max = 3) were computed on the inflection window (Bins 0.4–0.8) and full trajectory (Bins 0.1–0.9). Approximate p-values were derived from t-test transformation of the peak correlation coefficient, with effective sample size adjusted for lag (neff = n − |lag|). The inflection window was selected to encompass the complete PTGDS biphasic trajectory from compensatory peak to exhaustion. All analyses are fully reproducible using R v4.3.2 with set.seed(42); analysis code and intermediate outputs are deposited at https://github.com/YoungOukKim/MCI-to-AD.

### Segmented regression and change-point analysis

Segmented regression was performed using the segmented R package [34] with initial breakpoint estimate at 0.5; Davies’ test assessed breakpoint significance. Change-point estimation was conducted independently of ADNI clinical staging (Supplementary Fig. S1). Quadratic regression was performed at the donor level (n = 84 donors); cell-level testing on 67,419 nuclei inflates significance and was not used for inference. Model comparison employed ANOVA and AIC criteria. Peak location derived from the vertex (−β1 / 2β2). LOESS smoothing applied independently to visualize trend consistency.

### Computational analysis of neuronal vulnerability

Intercellular signaling was inferred using CellChat (v1.6.1) [35], focusing on LCN2–SLC22A17 interactions. Neuronal stress responses were quantified using SCENIC (v1.2.4) [36] for ATF3 and EGR1 regulon activity. Modules analyzed included NF-κB Priming (PTGS2, IL6ST, NFKBIA), Purinergic/Ca^2+^ Sensing (P2RY1, P2RY12, GJA1, ITPR2), Metabolic Buffering (HMOX1, SOD2, MT1E, MT2A, CLU, SLC1A2), and Immediate Early Stress (FOS, JUN, EGR1, ATF3). Detailed gene modules and intermediate signaling analyses are described in Supplementary Fig. S2.

### Reversible zebrafish MCI model

Zebrafish (Danio rerio, AB strain) [37,38] were maintained at 28.5°C (14:10 h light:dark). A chronic triple-stressor paradigm (LPS 5 μg/L, D-galactose 0.2 mg/L, 10% lard-based high-fat diet [39,40]) was applied from 2–14 dpf. Survival rate was ≥95% across all groups, with 30–45% cognitive impairment confirmed by the red ball avoidance task. Cognitive assessment was performed at 14 dpf using the red ball avoidance paradigm (Zefit Inc. protocol) [41,42]. Conservation of NF-κB binding motifs and orthologous ptgdsb.1/2 status were verified [43,44]. Brain tissue was collected at 14 and 21 dpf for qPCR (ptgdsb.1/2, ngfr, bdnf; primer sequences in Supplementary Table S2).

### Pharmacological modulation and BV-2 microglial assay

BXP-101 is a standardized multi-component formulation containing honokiol [45], wedelolactone [46], and atractylodin [47] as principal active constituents. Chemical standardization is shown in Supplementary Fig. S3. BXP-101 (0.3–0.6 μg/ml) was co-administered during the pre-inflection window (6–14 dpf). BV-2 murine microglial cells (passages 8–15) were cultured in DMEM supplemented with 10% FBS at 37°C, 5% CO_2_. Cell viability was assessed by MTT assay after 24 h exposure to BXP-101. For anti-inflammatory assays, cells were pre-treated with BXP-101 for 1 h before LPS stimulation (1 μg/mL, 24 h). Nitric oxide production was quantified using Griess reagent, and NF-κB p65 nuclear translocation was measured by immunofluorescence (anti-p65 antibody, 1:200; secondary Alexa Fluor 488, 1:500). Images were acquired on a Zeiss LSM 880 confocal microscope and quantified using ImageJ (nuclear/cytoplasmic ratio). All experiments were performed in triplicate (Supplementary Fig. S4).

### Behavioral and molecular analyses

Behavioral assessments used EthoVision XT 17 for zebrafish (locomotor and visual avoidance [41,42]) and mice (Y-maze [48], passive avoidance). General locomotor activity was assessed in a separate cohort (24-well plate, total distance moved) and is not included in the current analysis. Antibodies and primers used are listed in Supplementary Table S2.

### Y-maze and passive avoidance testing (murine)

The Y-maze was constructed from black polyvinyl plastic and consisted of three arms (40 cm in length, 4 cm in width, and 12 cm in height), positioned at 120° angles. Distinct visual cues were placed at the end of each arm. Each mouse was placed at one arm end and allowed to freely explore for 8 minutes. Spontaneous alternation (%) = [actual alternations / (total arm entries − 2)] × 100.

The passive avoidance test was performed in a light/dark apparatus (grid floor; 0.5 mA, 3 s foot shock). On Day 1 (acquisition trial), the latency to enter the dark compartment was recorded, followed by immediate foot shock delivery upon entry. On Day 2 (retention trial), mice were placed in the light compartment and the latency to enter the dark compartment was recorded with a cutoff time of 300 s.

### Mammalian validation

Male ICR mice (5 weeks; Koatech, Korea) received intracerebroventricular Aβ1–42 injection (20 μM) [49]. Groups: Sham, Aβ1–42, Aβ1–42 + BXP-101 (50/100/200/400 mg/kg), Aβ1–42 + Donepezil (5 mg/kg) (n = 10/group; final n = 9 for Aβ1–42 group after exclusion of one statistical outlier; Supplementary Table S3). Animals were housed at 23 ± 2°C, 50 ± 10% humidity, 12 h light/dark cycle with *ad libitum* access to standard diet (2018S; Envigo) and water.

### Network pharmacology and molecular docking

Target prediction used SwissTargetPrediction [50], PharmMapper [51], and DisGeNET [52], with supplementary databases OMIM [53] and GeneCards [54]; PPI network construction via STRING [55] in Cytoscape [56]; pathway enrichment via Metascape [57]. Molecular docking with AutoDock Vina [58] (exhaustiveness = 32); key targets: NF-κB p65 (PDB: 1NFI), GSK3B (PDB: 1Q3D), PTGS2 (PDB: 5KIR). ADMET profiles were predicted using pkCSM and SwissADME.

### ADNI CSF proteomics

ADNI CSF proteomics data comprised Emory TMT-MS and SomaScan 7K platforms (SeqIds: PTGDS X10514–5, LCN2 X2836–68, NEFL X10082–251). Clinical variables (MMSE, MoCA, diagnosis, age, sex, education, APOE ε4 [59]) were obtained from ADNIMERGE; CSF Aβ42, total Tau, and pTau181 from Roche Elecsys assays (UPENNBIOMK dataset). Protein abundances were log-transformed and per-batch z-normalized (batch = TMT plex / assay run) [60]. The NEFL neuronal-injury marker served as a positive control and was required to track the pathology axis (|r| > 0.3) before PTGDS or LCN2 were interpreted. Associations with cognition (MMSE) and pathology (log_2_[pTau181/Aβ42]) were quantified by Pearson correlation on each platform independently, reporting all coefficients. NEFL and PTGDS/LCN2 cognitive inflection points were estimated by segmented regression (Davies’ test); platform trend curves were visualised by locally weighted smoothing [61]. Confidence intervals for breakpoint locations were derived from the standard error of the estimated breakpoint (estimate ± 1.96 × SE; segmented package). Conversion was evaluated using a pre-specified, batch-corrected log-ratio restricted to baseline-MCI participants (Cox proportional hazards), reporting all results.

### Independent brain bulk proteomic validation (ROSMAP and Banner cohorts)

To assess whether the astrocytic PTGDS signal observed in single-nucleus and CSF data was independently detectable at the brain tissue protein level, we examined the published consensus tandem mass tag mass spectrometry (TMT-MS) AD proteomic dataset of Johnson et al. [62], which integrates 488 dorsolateral prefrontal cortex (DLPFC) tissues from the Religious Orders Study and Memory and Aging Project (ROSMAP, n = 84 control, 148 asymptomatic AD [AsymAD], 108 AD) and the Banner Sun Health Research Institute (n = 26 control, 58 AsymAD, 92 AD), profiled across 8,619 proteins. PTGDS- and LCN2-specific differential abundance results, Holm-adjusted p-values, and weighted gene co-expression network analysis (WGCNA) module assignments were extracted from Supplementary Table 2 of that study. To evaluate brain region generalization across the temporal-lobe regions interrogated by the SEA-AD MTG atlas, synthetic eigenprotein values for the PTGDS-containing module (M25 Sugar Metabolism) were obtained from Supplementary Table 17, which reports module-level statistics across ROSMAP BA-37 (inferior temporal cortex), Mt. Sinai Brain Bank BA-36 (parahippocampal gyrus), ROSMAP BA-6 (frontal cortex), and Emory BA-9/BA-24 cohorts. No reanalysis of raw spectra was performed; reported statistics are taken directly from the source publication.

### Statistical analysis

Analyses used R (v4.3.2) and Prism 10. Tissue- and donor-level associations were assessed by Spearman rank correlation. Mixed-effects models were implemented using lme4 [63]; time-to-event analyses used Kaplan–Meier estimation [64] and Cox proportional-hazards regression [65], with Benjamini–Hochberg FDR correction [66] where applicable. Data are mean ± SEM; p < 0.05 was considered significant. For murine behavioral assays, Y-maze data were analyzed by one-way ANOVA with Newman–Keuls post hoc, and passive avoidance data by two-way ANOVA with Bonferroni post hoc.

## Results

### A reproducible astrocytic inflection accompanies the compensatory-to-vulnerable shift

**Fig. 1.**
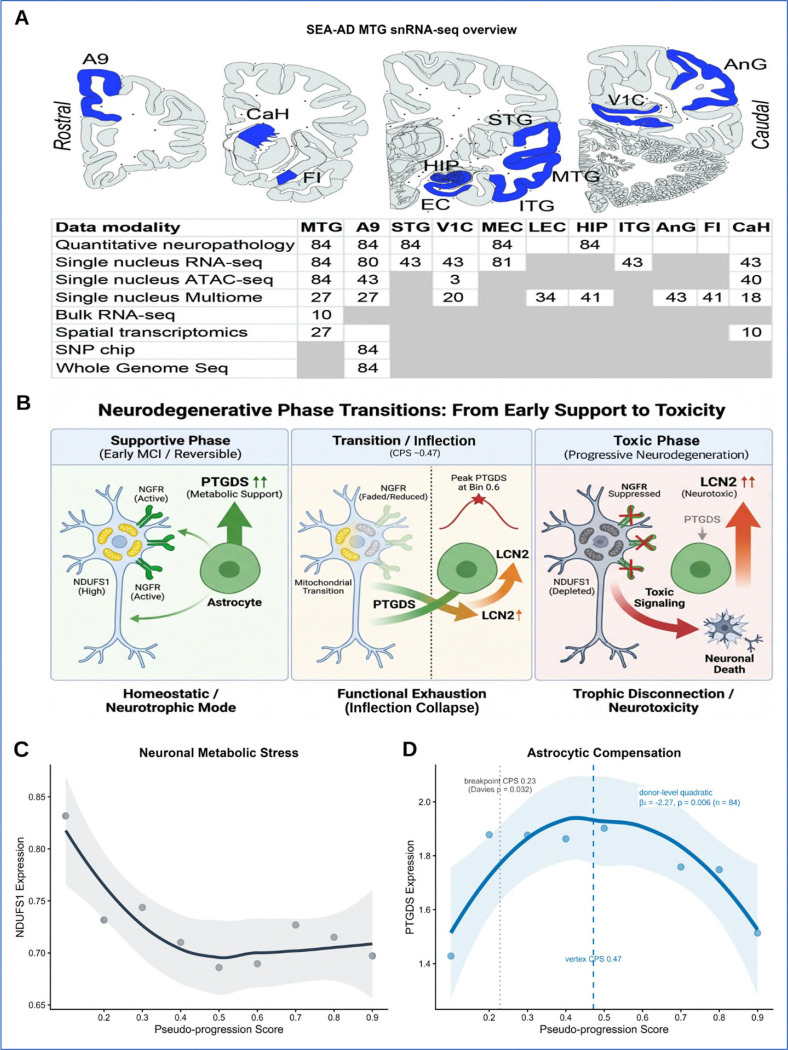
Single-nucleus trajectories in SEA-AD reveal an astrocytic PTGDS inflection at CPS 0.47. **A.** Study schema integrating SEA-AD snRNA-seq (1.3M nuclei, n=84 donors), reversible zebrafish MCI model, and ADNI CSF proteomics. **B.** Mechanistic framework (schematic): PTGDS-LCN2-NGFR axis from homeostatic buffering to neurotoxic signaling. **C.** Neuronal NDUFS1 decline from Bin 0.1; concurrent compensatory PTGDS upregulation (Table 1). **D.** Biphasic PTGDS trajectory (astrocyte, real expression): significant slope change at the segmented breakpoint Bin 0.23 (Davies’ p=0.032), with raw expression peaking across the CPS 0.5–0.6 bins; donor-level quadratic modelling places the curvature vertex at CPS 0.47 (β_2_=−2.27, p=0.006; donor n=84), with a significant post-peak decline (p=0.003) and a non-significant pre-peak rise. Data are mean±SEM. *p<0.05.

Analysis of 1.3 million nuclei from the SEA-AD atlas (84 donors; Fig. 1A) identified a reproducible astrocytic inflection associated with the compensatory-to-vulnerable shift during the MCI-to-AD continuum (Fig. 1A–D). By projecting nuclei onto CPS, we established a stage-resolved neurodegenerative trajectory framework (Fig. 1B) and identified a reproducible astrocytic trajectory centered on PTGDS dynamics. The earliest detectable event was neuronal NDUFS1 decline beginning at Bin 0.1, a core mitochondrial complex I subunit consistent with its established role as an early site of vulnerability during MCI and AD progression (Fig. 1C; Table 1) [67,68]. Astrocytic PTGDS expression increased in parallel and exhibited immediate inverse synchronization with neuronal NDUFS1 decline (Table 1), consistent with compensatory metabolic buffering — potentially through PTGDS-mediated prostaglandin signaling supporting astrocytic lactate production or anti-inflammatory buffering in response to neuronal energy deficit.

### Quantitative modeling supports a statistically resolved biphasic inflection

PTGDS expression demonstrated a robust biphasic trajectory supporting a statistically resolved neuropathological inflection (Fig. 1D; Table 2). Because single-cell counts (n = 67,419 astrocytes) inflate significance, all inferential statistics were computed at the donor level (n = 84). Donor-level quadratic modeling significantly improved fit relative to a linear model (β_2_ = −2.27, p = 0.006), confirming parabolic curvature with vertex at CPS 0.47. Stratified at CPS 0.47, expression showed a modest pre-peak rise followed by a steeper post-peak decline, indicating asymmetric dynamics of gradual compensation and accelerated decline. Segmented regression on donor-binned means (n = 9 bins) identified a significant early inflection at CPS 0.23 (Davies’ p = 0.032), marking the transition from compensatory upregulation toward deceleration prior to peak. Change-point estimation was performed independently of clinical staging (Supplementary Fig. S1).

### PTGDS inflection reflects astrocytic phenotypic reprogramming without evidence of cell loss

**Fig. 2.**
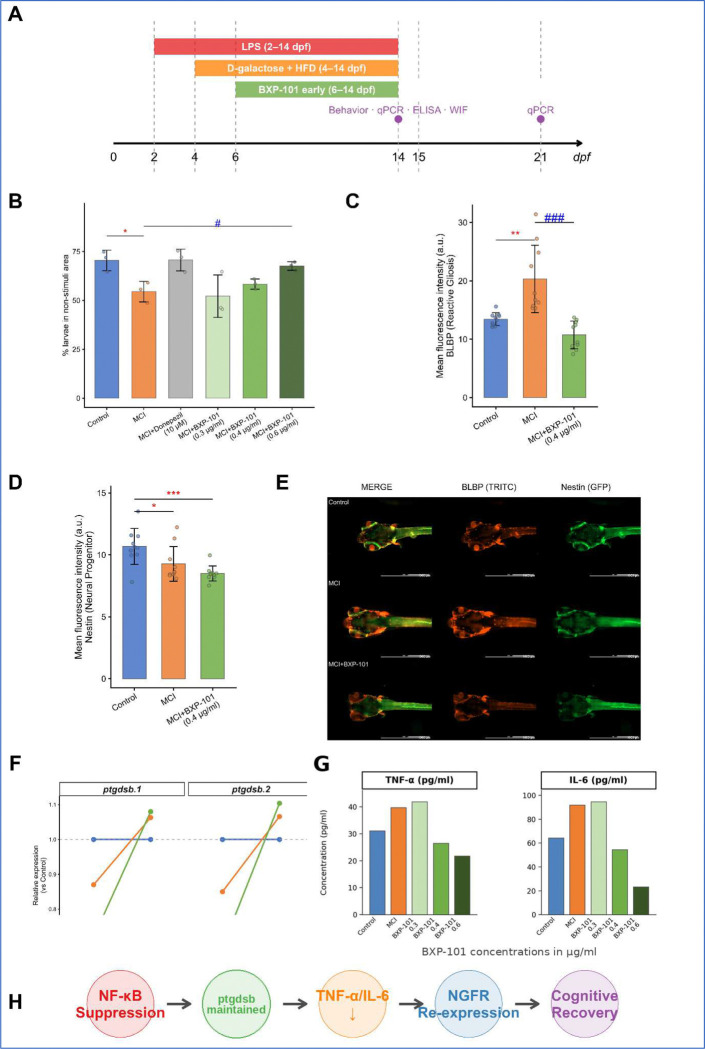
Recapitulation of the compensatory PTGDS induction phase and timing-dependent pharmacological modulation in a zebrafish MCI model. **A.** Experimental timeline: triple-stressor MCI induction (from 6 dpf); BXP-101 intervention during the pre-inflection window (6–14 dpf). **B.** Red ball avoidance at 14 dpf: MCI 22.7% reduction (70.5% to 54.5%; p=0.020 vs Control). BXP-101 0.6 μg/ml restored avoidance to 67.6% (p=0.016 vs MCI). Donepezil positive control: 70.7%. **C–D.** Whole-mount double immunofluorescence (BLBP/TRITC + Nestin/GFP) at 14 dpf: reactive gliosis (BLBP) and neural progenitor state (Nestin) quantified by mean fluorescence intensity (ImageJ, fixed ROI; n=10 larvae/group). **E.** Representative fluorescence images (Control / MCI / MCI + BXP-101 0.4 μg/ml). **F.** Longitudinal ptgdsb.1/2 qPCR at 14 and 21 dpf. ptgdsb.1/2 in MCI larvae remained near control, reaching modestly above control by 21 dpf (ptgdsb.1: 1.063±0.08; ptgdsb.2: 1.066±0.14), consistent with the early compensatory phase of the SEA-AD trajectory (CPS 0.1–0.47) and without the post-inflection decline. BXP-101 0.4 μg/ml maintained ptgdsb.1/2 near MCI levels (ptgdsb.1: 1.080 vs MCI: 1.063). BDNF: 1.71-fold vs control; PSD-95/dlg4: 1.27-fold vs control. **G.** ELISA (14 dpf): TNF-α −45% (MCI: 39.7 pg/ml; BXP0.6: 21.8 pg/ml); IL-6 −75% (MCI: 91.8 pg/ml; BXP0.6: 23.4 pg/ml). Anti-inflammatory effect more pronounced at protein than mRNA level, indicating post-transcriptional NF-κB modulation. **H.** Molecular recovery schematic: NFκB suppression → preserved ptgdsb.1/2 capacity → TNF-α↓ / IL-6↓ → NGFR re-expression. Data are mean±SEM. n=10 larvae/group (WIF); n=3 replicates/group (14 dpf qPCR); n=4 replicates/group (21 dpf qPCR). *p<0.05, **p<0.01, ***p<0.001 vs Control; #p<0.05, ###p<0.001 vs MCI.

Astrocyte abundance remained stable across Bins 0.4–0.8 (n = 41,141; 4,691–14,108 per bin), excluding population loss as the primary explanation for PTGDS decline. At the donor level (n = 84), astrocytes showed no evidence of apoptotic commitment: the anti-apoptotic regulator BCL2 increased with CPS (ρ = +0.30, p = 0.006), whereas pro-apoptotic CASP3 (ρ = −0.23, nominal p = 0.04) and BAX (ρ = −0.13, n.s.) did not increase (Supplementary Table S5). Astrocyte identity markers were largely preserved (SLC1A2 ρ = −0.02, n.s.), with a marginal decline in AQP4 (ρ = −0.21, p = 0.05). Across the CPS 0.5 boundary, astrocytes exhibited increased NF-κB-associated programs and gliosis markers (Supplementary Fig. S2), accompanied by reduction of ferroptosis-protective modules (GPX4, FTH1, SLC7A11; Supplementary Table S6). LCN2 transcripts were detected too sparsely in snRNA-seq (0.007%; 5/67,419 nuclei) for quantitative interpretation, so all LCN2-related interpretations rely on orthogonal validation (Fig. 2; Supplementary Fig. S5). Together, these findings indicate coordinated astrocytic state modulation accompanying PTGDS inflection, independent of astrocyte population loss.

### Exploratory lagged-correlation analysis suggests a directionally consistent astrocyte-neuron-microglia ordering across the PTGDS inflection window

**Fig. 3.**
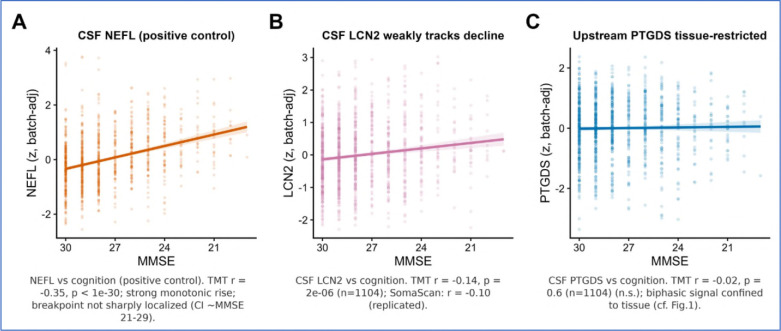
CSF concordance of the downstream effectors of the astrocytic PTGDS–LCN2 axis (ADNI; TMT-MS and SomaScan). **A.** CSF NEFL (positive control) rises strongly and monotonically with cognitive decline (TMT r = −0.35, p < 10^−30^); a discrete breakpoint is not sharply localized (95% CI ~MMSE 21–29). **B.** CSF LCN2 weakly tracks cognitive decline, most clearly on TMT-MS (r = −0.14, p = 2 × 10^−6^) where the NEFL positive control is satisfied; the SomaScan estimate is direction-consistent (r = −0.10) but supportive only, as its NEFL positive control does not clear the pre-specified |r| > 0.3 threshold, consistent with a downstream toxic effector. **C.** Upstream PTGDS shows no robust association with cognition (TMT r = −0.02, n.s.; n = 1104) and is only weakly correlated between platforms (r ≈ 0.15, below the threshold for cross-platform concordance; Supplementary Fig. S7), indicating the biphasic PTGDS signal is tissue-restricted (cf. Fig. 1). Abundances were log-transformed and per-batch z-normalized; NEFL served as a positive control. CSF measures provide mechanistic concordance, not stand-alone clinical prediction.

**Fig. 4.**
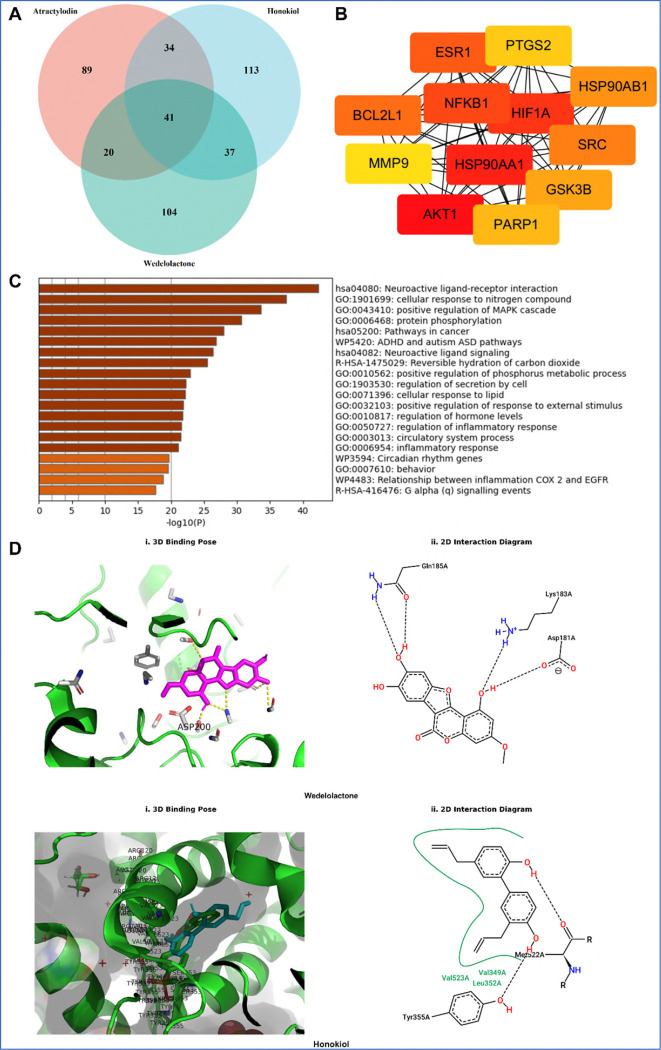
Network pharmacology and in silico molecular docking nominate NF-κB/GSK3B as candidate mechanistic hubs of BXP-101 (a multi-component NF-κB probe) action. **A.** Venn diagram of target overlap among atractylodin, honokiol, and wedelolactone. **B.** PPI network of top 12 hub targets. **C.** Top enriched pathways and GO terms (−log₁₀P). **D.** Representative docking poses: wedelolactone–GSK3B, honokiol–PTGS2. *p<0.05; Benjamini–Hochberg correction applied.

Within the PTGDS inflection window (Bins 0.4–0.8), lagged cross-correlation analysis revealed a directionally consistent ordering (Table 3; Supplementary Table S6). Purinergic/Ca^2+^ signaling covaried with PTGDS decline at a one-bin lag (Lag −1, r = −0.886), consistent with early compensatory sensing. NF-κB exhibited largely concurrent dynamics with PTGDS (Lag 0, r = −0.678). PTGDS decline was inversely associated with PPARG module expression (Lag 0, r = −0.775), indicating coordinated metabolic attenuation. Because astrocytic LCN2 transcripts were detected in only 0.007% of nuclei (5/67,419), the LCN2 arm of this cascade is not analyzable by snRNA cross-correlation; its placement downstream of PTGDS is instead supported by CSF proteomics (Fig. 3B) and cross-species qPCR (Figs. 2F, 4C). Microglial C3 showed an inverse correlation with neuronal NGFR (Lag 0, r = −0.940, p = 0.0176), consistent with astrocytes upstream and microglia as secondary amplifiers; however, neuronal NGFR transcripts are sparsely detected in snRNA-seq, so this association is exploratory and the NGFR arm is interpreted primarily from orthogonal qPCR (Fig. 2F) and murine data, not from snRNA abundance.

### Post-inflection destabilization reveals subtype-selective neuronal vulnerability

Comparison of excitatory neurons and SST^+^ inhibitory interneurons across CPS bins (Supplementary Fig. S6; Supplementary Table S7) demonstrated that while the PTGDS-LCN2-NGFR cascade was preserved across both neuronal classes, quantitative differences emerged. SST^+^ interneurons showed proportional reduction from 11.24% to 5.49% (as a fraction of excitatory plus SST neurons; donor-level Spearman ρ = −0.32, p = 0.003), with higher NDUFS1 expression variance (0.19–0.26 vs 0.12–0.16 in excitatory neurons), indicating increased metabolic heterogeneity under post-inflection stress. Excitatory neurons exhibited steep early NGFR decline (0.00476 to 0.00048, Bin 0.1→0.2), consistent with trophic signaling collapse, whereas SST^+^ neurons demonstrated more pronounced BCL2 reduction (6.8% decline, Bins 0.1–0.4) and elevated BAX/BCL2 ratio. These findings indicate a division of vulnerability roles: excitatory neurons primarily reflect trophic signaling collapse, while SST^+^ interneurons exhibit reduced apoptotic buffering capacity under PTGDS-associated metabolic stress.

### Recapitulation of the compensatory PTGDS induction phase and timing-dependent pharmacological modulation in a reversible zebrafish MCI model

In a reversible zebrafish MCI model (Fig. 2A), selective cognitive impairment was confirmed by a 22.7% reduction in red ball avoidance (70.5% to 54.5%; p = 0.020; Fig. 2B). Whole-mount double immunofluorescence (BLBP/TRITC + Nestin/GFP) revealed reactive glial dysfunction: BLBP fluorescence intensity was elevated 51% in MCI larvae (20.33 vs 13.43 a.u.; p &lt; 0.01; Fig. 2C–D), suppressed 47% following BXP-101 treatment (10.74 a.u.; p &lt; 0.001 vs MCI). Nestin signal intensity declined across MCI and treatment groups (MCI: 9.27 a.u.; BXP-101 0.4 μg/ml: 8.50 a.u.; Fig. 2E), consistent with reduced neural progenitor-associated activity under sustained inflammatory load. Longitudinal qPCR of ptgdsb.1 and ptgdsb.2 at 14 and 21 dpf showed that ptgdsb expression in MCI larvae was maintained near control levels, reaching modestly above control by 21 dpf (ptgdsb.1: 1.063 ± 0.08; ptgdsb.2: 1.066 ± 0.14; Fig. 2F) [43,44,69], without the pronounced post-inflection decline observed in the human atlas — consistent with the larval window capturing the early compensatory phase of the SEA-AD trajectory (CPS 0.1–0.47) rather than the later exhaustion limb. ngfr expression was concurrently reduced in MCI larvae [69], consistent with suppression of neurogenic signaling under sustained inflammatory load. Zebrafish ptgdsb.1/2 show 85.6% amino acid identity and high structural conservation with human PTGDS (RMSD &lt; 1.3 Å), with conserved NF-κB promoter motifs (Supplementary Fig. S5).

### Timing-dependent pharmacological modulation indicates a modifiable window at the metabolic inflection

**Fig. 5.**
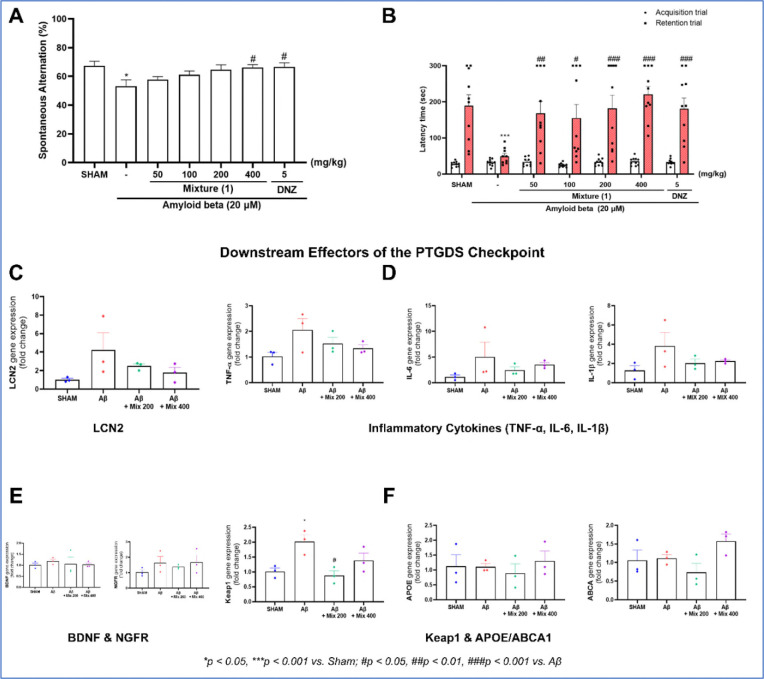
BXP-101, a broad NF-κB probe, modulates cognitive and molecular phenotypes in Aβ1–42 mice. **A–B.** Dose-dependent restoration of Y-maze spontaneous alternation and passive avoidance latency. **C–D.** Suppression of astrocytic LCN2 and pro-inflammatory cytokines (TNF-α, IL-6, IL-1β). **E.** Preservation of neuronal NGFR and BDNF. **F.** Normalization of Keap1; upregulation of APOE and ABCA1. Data are mean±SEM (n=8–10/group). One-way ANOVA + Tukey post-hoc. *p<0.05 vs Sham; #p<0.05 vs Aβ1–42.

Intervention during the pre-inflection window (6–14 dpf) significantly attenuated cognitive deficits (BXP-101 0.6 μg/ml: 67.6% vs MCI 54.5%; p = 0.016; Fig. 2B), consistent with pharmacological responsiveness during the compensatory phase preceding the CPS 0.47 inflection. BXP-101 reduced upstream inflammatory demand on PTGDS, evidenced by dose-dependent suppression of TNF-α (MCI: 39.7 pg/ml; BXP0.6: 21.8 pg/ml, −45% vs MCI) and IL-6 (MCI: 91.8 pg/ml; BXP0.6: 23.4 pg/ml, −75% vs MCI) at the protein level (ELISA; Fig. 2G). This anti-inflammatory effect was more pronounced at the protein than the mRNA level, suggesting post-transcriptional regulatory mechanisms consistent with the known modes of action of honokiol and wedelolactone as NF-κB modulators. BXP-101 treatment additionally suppressed reactive gliosis (BLBP: BXP0.4 10.74 vs MCI 20.33 a.u., −47%; p &lt; 0.001; Fig. 2C–D), and ptgdsb.1/2 levels at 21 dpf comparable to or marginally above MCI (ptgdsb.1: 1.080 vs MCI: 1.063; Fig. 2F), consistent with a demand-conservation interpretation in which NF-κB suppression reduces inflammatory burden on astrocytic PTGDS buffering. BDNF expression was elevated (1.71-fold vs control) and synaptic integrity partially restored (PSD-95/dlg4: 1.27-fold vs control; Fig. 2F) [70,71]. Network pharmacology analysis identified 12 hub targets enriched in neurogenesis and NF-κB pathways, with in silico molecular docking nominating GSK3B engagement (predicted binding affinity −8.3 kcal/mol; Fig. 4A–D). Behavioral assessments used the red ball avoidance paradigm (n = 30/group) [41,42] and Y-maze [48]. Murine validation (Fig. 5A–F; Supplementary Fig. S4) confirmed concordant dose-dependent effects on LCN2, NGFR, and inflammatory markers. Collectively, these data indicate that NF-κB modulation during the pre-inflection window is associated with preserved PTGDS-centered trajectory dynamics, providing functional support for the modifiability of the CPS 0.47 inflection. BXP-101 is used here as a broad, multi-component NF-κB probe rather than a PTGDS-specific intervention; these effects therefore indicate that the window is pharmacologically modifiable but do not by themselves establish PTGDS as the causal node (see Discussion).

### Downstream effectors NEFL and LCN2, but not PTGDS, track cognitive decline in ADNI CSF

CSF PTGDS is a constitutively secreted lipocalin reflecting astrocytic synthetic activity [32]; we therefore asked whether the tissue-level axis is detectable in CSF. Using two orthogonal ADNI platforms (TMT-MS and SomaScan) with per-batch normalization and NEFL as a positive control (NEFL vs pathology axis r = +0.48 [TMT], clearing the pre-specified |r| > 0.3 threshold; +0.24 [SomaScan], which does not clear it), we found that the downstream injury arm of the axis reaches CSF: NEFL — the strongest CSF signal and our positive control — rose strongly and monotonically with cognitive decline (r = −0.35, p < 10^−30^; Fig. 3A), although a discrete injury breakpoint was not sharply localized (95% CI spanning Mini-Mental State Examination (MMSE) [72] scores ~21–29). CSF LCN2 weakly tracked cognitive decline, most clearly on TMT-MS where the NEFL positive control is satisfied (TMT r = −0.14, p = 2 × 10^−6^); the SomaScan estimate was direction-consistent (r = −0.10) but is reported as supportive rather than confirmatory, because its NEFL positive control did not clear the pre-specified threshold (Fig. 3B) — consistent with LCN2 acting as a downstream toxic effector. In contrast, upstream PTGDS did not translate to CSF — it was only weakly correlated between platforms (r ≈ 0.15, below the threshold for cross-platform concordance; Supplementary Fig. S7) and showed no robust association with cognition (Fig. 3C) — indicating that the PTGDS biphasic signal is tissue-restricted (Fig. 1). In a pre-specified baseline-MCI analysis, neither batch-corrected PTGDS nor the PTGDS/LCN2 ratio predicted conversion to AD (PTGDS HR 1.04, p = 0.59; ratio HR 0.93, p = 0.37; n = 397 baseline-MCI, 177 converters; participant demographics in Supplementary Table S8); accordingly, the CSF data provide mechanistic concordance for the downstream effectors rather than a stand-alone predictive biomarker. Pseudo-time analysis independently recapitulated biphasic PTGDS dynamics with LCN2 induction at CPS 0.5–0.6 (Supplementary Fig. S2). Collectively, these observations converge on a reproducible astrocytic PTGDS inflection whose downstream inflammatory and injury effectors are weakly mirrored in CSF.

### Independent bulk-tissue proteomic confirmation of PTGDS upregulation (ROSMAP/Banner)

Single-nucleus and CSF data alone leave open the possibility that the observed PTGDS dynamics reflect transcriptional or secretion-level phenomena that do not propagate to bulk brain protein abundance. To address this, we examined the published consensus TMT-MS AD proteomic network of Johnson et al. (n = 488 DLPFC tissues from ROSMAP and Banner cohorts; 8,619 proteins) [62]. At the individual-protein level, PTGDS was significantly elevated in AD relative to control (mean log_2_ difference = +0.090; ANOVA F = 5.75, p = 3.4 × 10^−3^; Holm-adjusted p_AD–Control_ = 2.6 × 10^−3^; Supplementary Table S9), with a smaller, non-significant elevation in AsymAD (Holm-adjusted p = 0.17) and a further significant rise from AsymAD to AD (Holm-adjusted p = 0.046). This stage-resolved pattern—modest pre-symptomatic upregulation followed by additional elevation in clinical AD—is directionally consistent with the compensatory phase of the SEA-AD trajectory (CPS 0.1–0.47), recognizing that ROSMAP/Banner clinical AD cases distribute heterogeneously around the inflection point and that bulk tissue measurements integrate the full astrocytic compensatory load. PTGDS was assigned to the M25 Sugar Metabolism module (kME = 0.42), a co-expression community that includes metabolic proteins such as fatty acid synthesis (FASN, Holm p = 1.4 × 10^−6^), isoprenoid biosynthesis (GGPS1), acetyl-CoA metabolism (ACSS2), pentose phosphate flux (PGD, Holm p = 4.3 × 10^−9^), and SAM-cycle methylation (AHCY)—broadly metabolic in character. PTGDS is, however, a peripheral member of this module whose highest-connectivity hubs are cytoskeletal and transport proteins and several of whose members are not individually significant; the robust and reproducible signal in this dataset is therefore the elevation of PTGDS itself, not a coordinated module-level change. At the module level, the M25 synthetic eigenprotein was significantly elevated in AD relative to control specifically in ROSMAP inferior temporal cortex (BA-37; ANOVA p = 4.8 × 10^−3^; Tukey p = 3.2 × 10^−3^) and Emory anterior cingulate (BA-24; ANOVA p = 6.6 × 10^−3^), with a marginal trend in ROSMAP frontal cortex (BA-6; ANOVA p = 0.074). Module-level upregulation was thus regionally restricted (significant in 2 of 5 regions tested; the temporal BA-36 region was not significant, and the largest effect occurred in anterior cingulate rather than temporal cortex); the significant inferior temporal (BA-37) signal overlaps the temporal lobe sampled by SEA-AD but does not by itself establish temporal-lobe specificity. LCN2 protein showed a directionally concordant but non-significant elevation in the same dataset (mean log_2_ difference = +0.093, AD vs control; ANOVA F = 1.48, Holm-adjusted p = 0.29; Supplementary Table S9), with comparable effect size to PTGDS but markedly larger between-case variance, consistent with the inducible, threshold-gated kinetics expected of an NF-κB-regulated inflammatory amplifier and with the secreted nature of LCN2—a property that motivated the orthogonal CSF, qPCR, and murine validation already presented (Figs. 2F, 4C, 5). Together, these independent bulk-tissue observations demonstrate that the astrocytic PTGDS signal identified in single-nucleus and CSF data is reproducible at the brain protein level in two independent cohorts (ROSMAP, Banner) across the disease-relevant cortex examined.

## Discussion

By integrating single-cell pseudo-progression with cross-species validation, we characterize a temporally ordered astrocyte–neuron cascade that is structured around a statistically bounded metabolic inflection during the MCI-to-AD transition. As used herein, ‘transition boundary’ denotes a statistically defined inflection interval characterized by asymmetric trajectory dynamics and cross-scale conservation; it does not imply a strict thermodynamic discontinuity. Trajectory modeling revealed marked asymmetry: compensatory dynamics accumulated gradually, whereas post-inflection destabilization accelerated disproportionately, consistent with progressive compensation followed by accelerated vulnerability once metabolic buffering capacity is exceeded. This framework is supported by: (i) pseudo-progression–based directional relationships (Table 1, Table 3), (ii) pathway-level dissection of regulatory modules (Supplementary Fig. S2, Supplementary Fig. S4), and (iii) functional validation across zebrafish and murine models (Fig. 2, Fig. 5).

Three convergent observations support a directionally consistent PTGDS–LCN2–NGFR coupling framework. The limited statistical power of lagged CCF analysis (neff = 3–5) precludes definitive causal inference from correlation coefficients alone; however, the directional consistency of cascade ordering — combined with pharmacological modulation and cross-species validation — supports the proposed temporal hierarchy as a biologically coherent framework. First, purinergic sensing precedes PTGDS decline (Lag −1, r = −0.886), consistent with early compensatory mechanisms. Second, the placement of LCN2 downstream of PTGDS decline is supported by CSF proteomics and cross-species qPCR rather than snRNA cross-correlation, because astrocytic LCN2 transcripts are detected in only 0.007% of nuclei and are not analyzable at the transcript level. Third, microglial C3 inversely correlates with neuronal NGFR (Lag 0, r = −0.940, p = 0.0176), positioning astrocytes upstream in the observed cascade, with microglia acting as secondary amplifiers.

PPARG module changes were inversely rather than sequentially related to PTGDS and LCN2 trajectories, suggesting coordinated metabolic attenuation rather than a strict upstream regulatory role. While lagged correlations showed directional consistency in the core cascade, statistical significance was limited in several pairs due to small effective sample sizes after lagging (neff = 3–5). Early neuronal mitochondrial vulnerability, reflected by NDUFS1 decline (Table 1), coincides with compensatory astrocytic PTGDS upregulation. Following the inflection at CPS 0.47, PTGDS decline is associated with downstream LCN2 elevation (supported at the protein and CSF level; Fig. 3) and suppression of neuronal NGFR. Microglial activation follows this astrocytic shift (Table 3), positioning astrocytes upstream in the observed cascade, with microglia acting as secondary amplifiers [73]. Subtype analysis further reveals hierarchical neuronal vulnerability (Supplementary Fig. S6): SST^+^ interneurons show proportional reduction (11.24% to 5.49% of excitatory plus SST neurons; donor-level ρ = −0.32, p = 0.003; Supplementary Table S7) and increased metabolic variance, consistent with early excitation–inhibition imbalance [16] preceding circuit destabilization.

Neuronal NDUFS1 decline reflects early mitochondrial stress [67,68]. Within astrocyte–neuron metabolic coupling [74], PTGDS functions as a homeostatic effector [70]: its gradual compensatory induction may represent an attempt to maintain lipid-metabolic homeostasis under increasing energetic demand. Rather than a passive biomarker response, this pattern is consistent with an active buffering process whose attenuation coincides with the observed inflection. By analogy to tipping-point dynamics in complex systems [75], the PTGDS inflection is suggestive of a regime shift: prior to CPS 0.47, trajectories remain compatible with compensatory recovery, whereas beyond it, LCN2-mediated inflammatory amplification is associated with a shift toward accelerated vulnerability. We note that this dynamical-systems analogy is interpretive; cross-sectional pseudo-progression data cannot formally distinguish a true bifurcation from a steeper-than-linear gradient. This architecture — gradual compensatory loading followed by a steep inflection — is reminiscent of metabolic transitions described in oncology and cardiac remodeling [76], suggesting a potentially conserved principle of cellular stress tolerance across disease contexts.

Importantly, this inflection does not reflect astrocyte loss. Astrocyte abundance remains stable across bins 0.4–0.8 (n = 41,141), and correlations with apoptotic signatures are negligible (Supplementary Table S5). Instead, astrocytes shift toward a reactive, NF-κB-associated phenotype across the CPS 0.47 boundary, although the GFAP increase does not reach significance at the donor level (ρ = +0.10, n.s.; Supplementary Table S5), indicating phenotypic reprogramming rather than population collapse.

Module-level analysis (Supplementary Fig. S2) identifies layered regulatory architecture. Purinergic and calcium-associated genes (P2RY1, GJA1) precede PTGDS induction (Table 3), while buffering modules (HMOX1, CLU) shift from acute to sustained compensation. NF-κB activation was concurrent with PTGDS dynamics (Lag 0, r = −0.678; Table 3), suggesting that inflammatory priming and metabolic attenuation co-occur rather than following a strict sequential order. Instead, PTGDS-associated metabolic tone — potentially mediated through 15d-PGJ_2_–PPAR-γ signaling [77,78] — may transiently restrain downstream effector induction. Transition beyond CPS 0.47 coincides with LCN2 amplification and coordinated ferroptosis-related vulnerability [79,80].

Donor-level quadratic modeling confirms significant curvature (β_2_ = −2.27, p = 0.006) with a vertex at CPS 0.47 (Table 2), identifying a statistically resolved inflection interval. The asymmetry between a slow pre-peak rise and a steeper post-peak decline describes a tipping-point-compatible architecture rather than symmetric oscillation, though cross-sectional data cannot establish irreversibility per se. Together, these data are consistent with an inflection model in which the intervention window is shaped not by symptom severity alone but by position relative to the metabolic inflection point. Intervention before CPS 0.47 — corresponding clinically to approximately the MMSE 24–27 range — targets the compensatory phase, when metabolic buffering capacity is greatest and inflammatory amplification is not yet prominent. Intervention after this inflection may face a qualitatively different and less tractable target landscape.

LCN2 transcripts are detected in only 0.007% of astrocytes under baseline conditions, with the few detected transcripts appearing around the inflection — a pattern compatible with a threshold-like induction rather than a graded response. This relatively abrupt induction profile, reminiscent of ‘all-or-nothing’ inflammatory priming, is consistent with a steep inflection in the trajectory, though sparse transcript detection limits quantitative interpretation and motivates the orthogonal CSF and qPCR validation presented here.

Despite sparse LCN2 transcript detection in snRNA-seq (0.007%), consistent late-stage induction was supported across orthogonal platforms including CSF proteomics (Fig. 3), zebrafish qPCR (Fig. 2F), and murine models (Fig. 5C). Integration of ADNI CSF proteomics provides quantitative clinical alignment: CSF LCN2 weakly tracks cognitive decline across two platforms and NEFL rises strongly with cognitive decline (Fig. 3A–B), consistent with downstream effectors of the tissue-level CPS 0.47 inflection reaching CSF. This concordance links a cellular metabolic inflection to clinically measurable decline at the level of downstream effectors, without implying a stand-alone CSF predictor. Pharmacological suppression of LCN2 during the pre-inflection compensatory window was associated with restored NGFR expression and cognitive performance (Fig. 2H; Fig. 5), consistent with the interpretation that pharmacological responsiveness is constrained by molecular position relative to this metabolic inflection. While pseudo-time inference cannot establish definitive causality [71,75], convergence across 1.3 million nuclei, orthogonal perturbation models, and clinical proteomics supports the biological relevance of this inflection. This convergence is further reinforced at the brain bulk-tissue protein level by the published ROSMAP and Banner consensus TMT-MS dataset (n = 488 DLPFC tissues) [62], in which PTGDS itself is significantly elevated in AD (Holm-adjusted p = 2.6 × 10−3) and the PTGDS-containing M25 metabolism module—encompassing FASN, GGPS1, ACSS2, and PGD—is most strongly upregulated in inferior temporal cortex (BA-37; module-level p = 4.8 × 10−3; Supplementary Table S9), the temporal-lobe region anatomically matched to the SEA-AD MTG atlas.

LCN2 induction occurs within a broader remodeling of lipid-associated pathways. APOE and CLU display distinct but complementary trajectories (Table 1), indicating partial redundancy within lipocalin-associated buffering systems. As PTGDS declines, LCN2 induction aligns with iron-linked inflammatory signaling, suggesting a qualitative shift in astrocytic metabolic state.

Lagged cross-correlation analysis (Table 3) shows that PTGDS decline is followed by PPARG module attenuation (Lag 0, r = −0.775), consistent with weakening anti-inflammatory restraint. The LCN2 arm could not be assessed by snRNA cross-correlation (transcript detected in only 0.007% of astrocytes); instead, CSF and bulk-tissue protein data (Fig. 3; ROSMAP/Banner) together with cross-species qPCR are consistent with astrocyte-derived LCN2 acting as a secreted mediator that propagates metabolic stress to neurons. LCN2 is known to induce iron dysregulation and ferroptosis sensitivity [79,80], consistent with the mitochondrial complex I vulnerability reflected by NDUFS1 decline, and to impair neurotrophic signaling pathways (NGFR downregulation). Together, these correlational data are consistent with a temporally ordered coupling model in which astrocytic metabolic reprogramming precedes neuronal vulnerability, while acknowledging that pseudo-temporal ordering alone cannot establish causal directionality.

Several limitations warrant consideration. First, SEA-AD provides pseudo-time trajectories rather than true longitudinal sampling, limiting causal inference. Although lagged cross-correlation and timing-dependent perturbation experiments support directional interpretation, definitive causality requires targeted perturbation in human-relevant systems. Relatedly, donor age at death was only weakly and non-significantly correlated with CPS (Spearman ρ = −0.14, p = 0.19, n = 84 donors; Supplementary Fig. S8), indicating that the inflection describes a disease-progression boundary within a uniformly aged cohort (median age 90 years) rather than an age-driven change per se; fully disentangling the contributions of chronological aging and disease progression would nonetheless benefit from age-stratified or longitudinal designs.

Second, LCN2 transcript detection in droplet-based snRNA-seq was sparse (0.007%; 5 astrocytes among 67,419), a known limitation for low-abundance or secreted transcripts. Conclusions regarding LCN2 trajectory rely on orthogonal validation platforms, including CSF proteomics and cross-species qPCR, which consistently supported pathway engagement.

Third, the SEA-AD dataset is restricted to the middle temporal gyrus (MTG); thus, our findings may not fully capture the regional heterogeneity of AD progression. Future studies integrating spatial transcriptomics or multi-region atlases will be essential to validate these metabolic inflections across differentially affected brain areas.

Fourth, cross-species validation in zebrafish was performed in larvae (≤21 dpf), a developmental window that captures the early compensatory limb of the PTGDS trajectory but does not extend to the post-inflection exhaustion phase resolved in the aged human atlas. Accordingly, the zebrafish data support conservation of the compensatory induction phase and provide a tractable system for timing-dependent pharmacology, but do not by themselves recapitulate the full biphasic trajectory; resolving the descending limb will require aged adult zebrafish (≥6 months), in which age-associated PTGDS dynamics can be followed beyond the compensatory window.

Fifth, several interpretive caveats apply to the inflection itself. We use the term “inflection” to describe a statistically resolved change in trajectory dynamics, supported convergently by segmented regression, quadratic modeling, and pseudo-time analysis. While the consistency of these complementary approaches reduces the likelihood that the inflection is a single-method fitting artifact, cross-sectional pseudo-progression data cannot formally establish that the underlying biology constitutes a true dynamical-systems bifurcation as opposed to a steeper-than-linear but continuous gradient; the dynamical-systems language used here should therefore be read as an interpretive analogy rather than a formal claim. Relatedly, the clinical alignment we report should be understood as a probabilistic risk regime rather than a hard threshold: the MMSE values reported (crossover near 26, with a 95% confidence interval of approximately 24–27) represent the centroid of a range derived from a noisy clinical instrument with appreciable test–retest variability, and individual trajectories are expected to distribute around, rather than switch precisely at, this window.

Finally, pharmacological modulation with BXP-101 attenuated deficits when applied during the pre-inflection compensatory window, consistent with the inflection model; whether this responsiveness is lost once the inflection is crossed — as the asymmetric trajectory would predict — remains to be tested directly. However, BXP-101 broadly modulates NF-κB signaling, and the observed anti-inflammatory effects — TNF-α suppression (−45%), IL-6 suppression (−75% at protein level) — are better interpreted as downstream correlates of upstream metabolic stabilization than as evidence of a primary mechanism. In this context, NF-κB activation may reflect amplification enabled by PTGDS exhaustion rather than a causal driver, although our data cannot establish this directionality definitively. More specific PTGDS-directed approaches — such as PGD_2_ analogs or conditional PTGDS restoration — will be required to test direct metabolic causality and to determine whether the trajectory can be reversed once the inflection is crossed.

Our findings identify astrocytic PTGDS inflection as a reproducible candidate stage marker associated with the shift from reversible prodromal compensation toward accelerated neurodegenerative progression in Alzheimer’s disease. The convergence of this inflection across 1.3 million single nuclei, orthogonal perturbation models, and longitudinal clinical proteomics — with cross-scale alignment in the MMSE 24–27 range — provides a quantitative framework relevant to stage-stratified intervention. The window of pharmacological responsiveness associated with this inflection appears to be determined not by symptom severity alone but by molecular position relative to the PTGDS inflection point. These findings suggest a shift in prodromal AD therapeutics from symptom-driven timing toward mechanism-anchored staging, with potential implications for clinical trial design and patient stratification.

The pre-inflection responsiveness observed with BXP-101 is consistent with the interpretation that the compensatory phase preceding PTGDS exhaustion remains amenable to modulation of the underlying energy-stress axis. Although the present study did not deconvolve the contributions of individual constituents, the three principal components of this formulation have each been independently reported to engage complementary nodes of this axis: atractylodin attenuates NF-κB–driven neuroinflammation and NLRP3 signaling while supporting BDNF/Akt activity [81]; wedelolactone modulates the Keap1–NRF2 antioxidant response [82], a pathway that itself acts as a negative regulator of NF-κB; and honokiol enhances mitochondrial bioenergetics through SIRT3 activation [83]. Read together with the convergent inflammatory, redox, and bioenergetic features of the inflection described here, these reported activities provide a plausible mechanistic rationale for targeting multiple nodes of a multi-component stress axis rather than a single effector. We emphasize, however, that the individual constituent contributions and any synergistic interactions were not experimentally separated in this study, and our pharmacological data should therefore be interpreted as a coarse-grained perturbation of this axis rather than as evidence for a specific molecular target. Dedicated constituent-resolved and target-specific studies will be required to establish the precise mechanistic basis of these effects.

## Supplementary Material

Supplementary Files

This is a list of supplementary files associated with this preprint. Click to download.

• SupplementaryDatafilewithS9.docx

• SupplementaryTableS9.xlsx

## Figures and Tables

**Table 1.** Cell-type-resolved expression trajectories in SEA-AD astrocytes and neurons across CPS bins

Marker (Cell Type)	Bin 0.1 (Early)	Bin 0.5–0.6 (Mid)	Bin 0.9 (Late)	Trajectory Characterization
NDUFS1 (Neuron)	0.7817	0.6980	0.7061	Overall declining trend from Bin 0.1
PTGDS (Astrocyte)	1.6531	1.9115	1.6310	Biphasic: slope shift at Bin 0.23; peak near CPS 0.47
NGFR (Neuron)	0.00262	0.00058	0.00073	Early decline with sustained low plateau
APOE (Microglia)	1.1820	1.1004	1.2186	U-shaped; mid-stage nadir

**Note:** Expression values represent 3-bin moving averages of log-normalized expression from the SEA-AD middle temporal gyrus atlas (1.3 million nuclei, 84 donors). Segmented regression on PTGDS identified a significant inflection at Bin 0.23 (95% CI: 0.13–0.33; Davies’ p = 0.032). LCN2 detection rate in astrocytes was 0.007% (5/67,419); all LCN2-related interpretations are supported by orthogonal datasets (ADNI CSF proteomics, zebrafish qPCR, murine qPCR). Detailed bin-resolved data are provided in Supplementary Table S4.

**Table 2.** Statistical validation of biphasic PTGDS dynamics at the donor level (n = 84 donors)

Component	Estimate	p-value	Interpretation
Quadratic term	β_2_ = −2.27	p = 0.006	Significant biphasic curvature (donor-level, n=84)
Vertex CPS	0.47	—	Peak inflection point
Pre-peak trend	positive	p = 0.45 (n.s.)	Compensatory rise (not significant at donor level)
Post-peak trend	negative	p = 0.003	Progressive decline (donor-level)
Segmented inflection	CPS 0.23 (95% CI: 0.13–0.33)	Davies p = 0.032	Early deceleration onset

**Note:** Quadratic and linear regression models fitted using CPS as a continuous variable at the donor level (n = 84 donors; cell-level testing on 67,419 nuclei was not used for inference). Vertex position derived from the quadratic model (−β1 / 2β2). Pre-peak: CPS ≤ 0.47, n = 25 donors; Post-peak: CPS > 0.47, n = 59 donors. Segmented regression performed on unsmoothed bin-level means (n = 9 bins) using Davies’ test.

**Table 3.** Lagged cross-correlation analysis within the PTGDS inflection window (Bins 0.4–0.8)

Interaction Pair	Cell Types	Lag (Bins)	r-value	Interpretation
Purinergic → PTGDS	Astro ↔ Astro	−1	−0.886	Sensing precedes compensation
NF-κB → PTGDS	Astro ↔ Astro	0	−0.678	Concurrent inflammatory association
PTGDS → PPARG	Astro ↔ Astro	0	−0.775	Coordinated metabolic attenuation
C3 → NGFR	Micro ↔ Neuron	0	−0.940*	Complement-mediated neurotoxicity

**Note:** Lagged cross-correlations computed on 3-bin moving average trajectories within the inflection window (Bins 0.4–0.8). Negative lag indicates source precedes target. Due to limited effective sample size after lagging (neff = 3–5), p-values are not reported except *p = 0.0176 for C3 → NGFR. Astrocytic LCN2 pairs were excluded because LCN2 was detected in only 0.007% of astrocytes (5/67,419); the LCN2 arm is instead supported by CSF proteomics and cross-species qPCR (Figs. 2F, 4C, 5). Full trajectory analysis in Supplementary Table S6.

## Data Availability

The single-nucleus RNA-sequencing data analysed in this study are available from the Allen Brain Cell Atlas (SEA-AD) at https://portal.brain-map.org. The ADNI CSF proteomics data are available through https://adni.loni.usc.edu upon completion of standard data use agreements. The ROSMAP and Banner consensus brain proteomic statistics used for independent validation were obtained from the published study of Johnson et al. [62] (Supplementary Tables 2 and 17); no raw spectra were reanalysed, and the underlying proteomic datasets are available through the AD Knowledge Portal (https://adknowledgeportal.synapse.org). All other data supporting the findings of this study are available within the article and its Supplementary Information files.

## Code availability

The custom analysis code used to generate the results reported in this study is available at https://github.com/YoungOukKim/MCI-to-AD.
